# Artificial intelligence and health empowerment in rural communities and landslide- or avalanche-isolated contexts: real case at a fictitious location

**DOI:** 10.3389/fdgth.2025.1655154

**Published:** 2025-08-26

**Authors:** Rune Johan Krumsvik, Vegard Slettvoll

**Affiliations:** ^1^ Faculty of Psychology, University of Bergen, Bergen, Norway; ^2^ Faculty of Medicine, University of Bergen, Bergen, Norway

**Keywords:** artificial intelligence, landslide, avalanches, isolated villages, rural healthcare, health empowerment, simulated rural setting, brief case report

## Abstract

Through a series of case studies, we have pretested the capabilities and reliability of the Large Language Models (LLM), Generative Pre-trained Transformer 4 (GPT-4) and OpenAI o3 reasoning model (o3) in educational and healthcare contexts. Based on this knowledge, we took a step further by testing these technologies in an authentic patient case set in a fictitious location. The context for this brief case report relates to the fact that, in the first quarter of 2025, fewer patients lacked an assigned GP compared to previous years—a positive trend. However, this offers little relief to those cut off from GP care due to their rural location or because of landslides and extreme weather. This case highlights the need for knowledge-based preparedness and alternative health empowerment pathways in rural Norway. This brief case report describes a single 16-year-old boy (*N* = 1) with no significant past medical history or chronic conditions. Although he lived in an urban area, we reframed the encounter as a simulated rural, avalanche-isolated scenario to test the feasibility of AI-supported care under extreme access constraints. Specifically, the case models how a patient in an avalanche-prone mountain valley—where seasonal road closures routinely sever access to healthcare facilities—could receive rapid, guideline-concordant treatment for severe tonsillitis during a period of general-practitioner (GP) unavailability. Repeated attempts to secure a same-day appointment were thwarted by workforce shortages and impassable roads, resulting in the earliest available appointment being five days away. The family leveraged point-of-care technologies (fingerstick C-reactive protein analysis, wearable sensors, blood pressure device, digital fever device, mobile ECG) and an o3 language model[1] to evaluate disease severity. A peak CRP of 130 mg/L, combined with otherwise stable vital signs, prompted a remote consultation with a trusted physician in their social network, who confirmed the diagnosis of bacterial tonsillitis and initiated treatment with phenoxymethylpenicillin (Apocillin). Within 72 h, CRP fell to 23 mg/L and symptoms were resolved. The patient case and the events described in this pilot study are authentic, but the location is fictitious. The waiting time to see a general practitioner was five days in both the actual urban setting and the simulated rural scenario; however, unlike in urban contexts—where patients can often access immediate care through emergency clinics or private GPs—such options are typically unavailable in sparsely populated rural areas. This case illustrates how AI and health technology can serve as a “virtual waiting room” for individuals in rural or landslide- and avalanche-isolated areas, especially when GP access is limited and the condition is low-risk, such as mild sore throat symptoms. The case illustrates how inexpensive diagnostics and AI-supported reasoning can strengthen health empowerment and temporarily bridge care gaps for residents of geographically isolated Norwegian communities—provided that human clinical oversight and robust digital health governance remain in place. Therefore, all LLM recommendations and technology support were reviewed during an in-person physician examination in a family network, and the final antibiotic prescription came from the clinician, underscoring that AI functioned solely as decision support rather than autonomous care.

## Introduction

Norway ranks among the world's leaders in healthcare personnel per capita, boasting the fourth-highest physician density in the The Organisation for Economic Co-operation and Development (OECD) (1). Although Norway's Regular GP Scheme is highly rated internationally, ongoing staffing shortages, long distances, and frequent natural hazards increasingly prevent timely access to care—particularly for residents in remote mountain valleys and fjord side villages prone to landslides and avalanches. Number of residents in sparsely populated rural areas are 924,262, which is roughly 17% of Norway's population (2). Maintaining a stable medical service in remote and sparsely populated areas has long been challenging—a reality well known across the Nordic countries. Recruitment difficulties of GPs are markedly greater in these rural districts (3). National surveillance showed that in the first quarter of 2025, 2.7 percent of the population did not have a regular doctor assigned to the patient list they are registered on. This marks an improvement since 2023, when the proportion reached 4 percent. Several counties have shown positive developments in 2024 (4, 5). This improvement is encouraging but offers little consolation to those who are unable to reach their GP due to their rural location or because their communities are cut off by landslides, avalanches, or flooding. It is therefore essential to build a knowledge-based preparedness for this kind of health empowerment in natural disaster scenarios—events that, unfortunately, are becoming increasingly common due to extreme weather in Western and Northern Norway. Only about 11% of Norway's general practitioners practice in municipalities with fewer than 5 000 inhabitants—even though such small municipalities (often with even smaller mountain villages, etc.) make up roughly half of all Norwegian municipalities (3).

Geographic isolation and extreme weather amplify capacity gaps. Rainfall and snow accumulation are important factors that influence the risk of landslides. In Norway, both the frequency and intensity of extreme precipitation events are already increasing, which contributes to a rise in landslides and debris flows caused by heavy precipitation (6). Recent developments show a notable increase in the number of landslide-related incidents in Norway. The Norwegian Soil and Water Database (NSDB) now includes over 97,000 recorded events—representing a sharp rise due to both improved modern reporting practices and increased events (7). Climate change plays a crucial role, with more frequent and intense precipitation events contributing to an increased incidence of both soil and rock landslides, particularly in vulnerable areas of Western and Northern Norway (8). In sparsely populated regions, the absence of robust infrastructure and limited emergency response capacity often results in isolation and delayed health support (and sometimes delayed evacuations) during such events (9). Qualitative studies and survey studies from Western and Northern Norway describe how winter avalanches, storm-related road closures, and seasonal ferry cancellations can strand entire settlements for days, interrupting all routine healthcare contacts and forcing residents to defer care or mobilize informal networks (10, 11). In a multicentre interview study spanning eight rural municipalities, frontline staff linked patient-safety incidents to locum turnover, work overload, and hazardous travel conditions such as avalanche-closed mountain passes (12). Could the use of AI, digital self-testing, and other digital tools—both in everyday life and during periods of isolation or acute illness—represent an untapped potential for strengthening rural health empowerment and healthcare?

Against this backdrop, consumer diagnostics and conversational artificial intelligence (AI) are being piloted as supplemental triage channels. A national evaluation of the digital symptom-triage form on **Helsenorge.no** found that the tool safely redirected 18 % of potential face-to-face appointments at five GP clinics during 2023–2024 (13). Complementing these local findings, a 2025 systematic review of 73 studies concluded that AI applications in primary care can enhance diagnostic accuracy and patient self-efficacy—provided robust governance and clinician oversight are in place (14). Other studies report comparable findings (15–22). The present case, set in a fictive avalanche-isolated mountain valley, exemplifies how such digital adjuncts based on *symbiotic intelligence* can preserve assessment continuity when conventional GP services are temporarily unreachable. *Symbiotic intelligence* (23, 24) refers to a clinician-supervised model of care in which home-collected biomarkers and continuous wearable data are integrated in real time with reasoning from an o3 chatbot (or comparable LLM), yielding joint human-AI decision-making that neither partner could achieve alone. It is closely linked to symbiotic design (25) and to the concept of symbiopersonal intelligence in healthcare (26).

Norway ranks among the global leaders in fixed broadband: 99.1% of households can obtain ≥100 Mbit/s, yet coverage drops to 94.7% in sparsely populated (“spredtbygd”) districts, where a single fibre or fixed-wireless link—readily severed by avalanches—may be the only lifeline (27). National figures for clinician supply show a formal density of 21 general practitioners per 10 000 inhabitants in local counties like Finnmark vs. 10.2 in in cities like Oslo, but the headline numbers obscure much longer travel times in the north, where surgeries are scattered across vast distances and roads are frequently closed by snow, ice, or landslides (28). During night-time hours or severe weather, remote municipalities must route urgent calls to inter-municipal out-of-hours hubs that can be many miles away and reachable only by telephone. These structural constraints intersect with a wider rural digital divide: older populations, lower household incomes, and limited AI literacy dampen demand for subscription-based services and point-of-care equipment. Comparable concerns have recently been taken up by the European Commission (29). Together, these factors illustrate why AI-supported care will remain conditional on robust connectivity, affordable devices, and readily available clinical oversight in non-urban settings.

Our own research at the University of Bergen's Digital Learning Communities Artificial Intelligence Centre (8) the language model GPT-4 has demonstrated strong performance on national examinations in medicine and nursing (in Norwegian language), and has shown the ability to provide high-quality formative feedback at the PhD level. This suggests significant potential for enhancing health literacy and promoting patient empowerment (30–36). Our ongoing pilot research [2] extends beyond classroom benchmarks to case pilots related to rural communities, and landslide and avalanche isolated municipalities. In these pilot studies, GPT 4 and o3 chatbots simulate a guiding of residents through symptom appraisal, wearable sensor interpretation, and basic triage when e.g., access to GP in everyday life or snow blocked roads cut off all professional care for several days. Nevertheless, robust evidence on safety, equity, and integration with Norway's Regular GP Scheme remains scarce — underscoring the need for real world examples like the present case that document AI supported care pathways in rural, hazard prone settings. The present case therefore addresses an unconventional and new management pathway for severe tonsillitis during a period of limited primary-care availability in a fictive rural setting. The research question is: *To what extent, and in what ways, might low-cost diagnostics and AI-supported decision-making contribute to health empowerment in managing severe tonsillitis in rural areas with limited access to primary care?*

## Case description

The patient case (*N* = 1) and the incidents in this pilot study are authentic, but the location is fictitious. The term “fictitious” signals that the clinical events and patient data are genuine, whereas the remote, sparsely populated location is an anonymized construct—used to explore feasibility in an avalanche-isolated setting that we could not practically access during this pilot. In reality, the patient lives in an urban area; however, the case has been contextualized as if it took place in a remote rural setting. This was done to illustrate how similar health challenges could be addressed in sparsely populated areas with limited access to medical services, thereby highlighting the potential of low-cost diagnostics and AI-supported care in such contexts. The waiting time to see a general practitioner was five days in both the actual urban setting and the simulated rural scenario; however, unlike in urban contexts—where patients can often access immediate care through emergency clinics or private GPs—such options are typically unavailable in sparsely populated rural areas.

**Figure 1:**
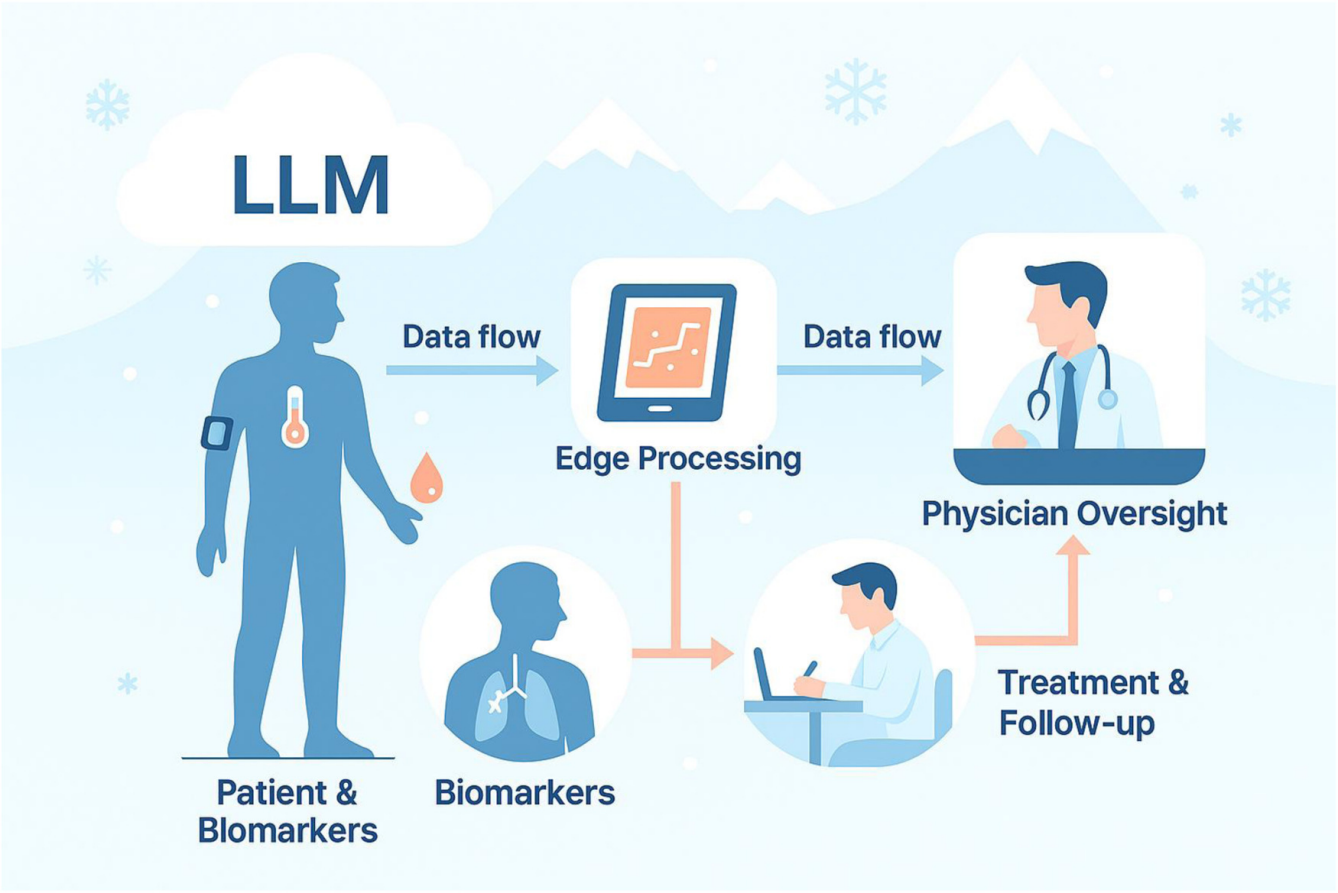
Symbiotic AI–supported care pathway for avalanche-isolated rural patients.

As an alternative in this case, a licensed physician in the family network performed an in-person examination and made the prescribing decision, while the LLM acted only as decision-support prompting escalation and red-flag vigilance. The patient had no significant past medical history or chronic conditions. Figure 1 illustrates the workflow of the process.

**Day 0 (Monday 28 April)** – A 16-year-old boy returned home from school presenting with a high fever of 39.5 °C. He reported a sudden onset of sore throat, difficulty swallowing, and a general feeling of malaise. Clinical signs included markedly swollen and erythematous tonsils, bad breath (foetor ex ore), and visible discomfort when attempting to eat or drink. Despite taking paracetamol (2 × 500 mg every 5 h), the medication provided minimal symptomatic relief. The persistent high fever and local symptoms raised concern for possible bacterial tonsillitis, mononucleosis, influenza or other viral infections. A preliminary, general prompt was directed to LLM o3 for a second opinion (Part 1).

A 16-year-old boy is sick with body aches, a high fever, and severe throat pain. His resting heart rate and blood pressure are normal. Could you try to make a diagnosis based on this information?

A condensed response from o3: *The numbers primarily point to acute bacterial tonsillitis (most likely streptococcal), but an early peritonsillar abscess must also be ruled if the CRP is high and the throat pain is severe. A medical evaluation with CRP, a throat swab (and possibly imaging) is necessary for a definitive diagnosis and appropriate treatment* (37).

**Day 1 (Tuesday 29. April)** – Cervical lymph-node swelling and odynophagia progressed; oral intake was restricted to liquids. Fever still at 39.5°C. To receive support from AI and guide the o3 language model toward a clinically relevant assessment, a more stringent chain-of-thought prompt (in Norwegian) was designed to clearly outline the patient's symptoms, context, and the central diagnostic challenge. This structured approach helped the model's reason step-by-step and arrived at a plausible interpretation:

You are a medically informed assistant helping to evaluate a 16-year-old boy who returned home from school with a high fever (39.5°C). He presents with a sore throat, swollen red tonsils, difficulty swallowing, and bad breath. Paracetamol (2 × 500 mg every 5 h) has provided little relief. He lives in a remote area where access to a doctor is currently unavailable due to road closures. Based on this clinical picture and context, what is the most likely diagnosis, and what would be an appropriate next step in management?

Preliminary analysis of the symptoms by using o3 (condensed, see attachment 1): *The data mainly point to acute bacterial tonsillitis (most likely streptococcal), but an early peritonsillar abscess must also be excluded because if the CRP is high and severe throat pain is lasting. A medical assessment with CRP and throat sampling (and possibly imaging) is necessary for a definite diagnosis and appropriate treatment* (37).

**Day 2 (Wednesday 30. April)** – Avalanched closed roads and repeated phone calls to the GP clinic were unsuccessful because of fully booked schedules and physician absences; the earliest available appointment was five days away. The patient's family had access to CRP analyzer, blood pressure device, digital fever device, ECG device for home use, advanced wearables and o3-subscription and long experience with preliminary health analysis with GPT-4 and o3. More specifically, this was a home finger-stick CRP measured 130 mg/L (reference <5 mg/L) using a Aidian QickRead Go analyzer. Fever was 39.7 °C (Microlife digital fever thermometer), blood pressure was 112/68 mmHg (Microlife), resting heart rate 58 bpm (Garmin Fenix 6X PRO Solar), and mobile ECG (KardiaMobile) showed normal sinus rhythm. AI-assisted triage—History, vital signs, and CRP were anonymously entered into a OpenAis most powerful reasoning model—the o3 language model (38).

An extended chain-of-thought prompt was created to reflect the full clinical and contextual complexity.

As mentioned, you are a medically informed assistant helping to evaluate a 16-year-old boy who returned home from school with a high fever (39.7 °C), sore throat, swollen and red tonsils, difficulty swallowing, and bad breath. Paracetamol (2 × 500 mg every 5 h) has provided little relief. The family lives in a remote, avalanche-prone mountain area where road closures have cut off access to healthcare. Repeated attempts to contact the local GP clinic were unsuccessful due to fully booked schedules and physician absences, and the earliest available appointment was five days away. The family has access to point-of-care diagnostic tools at home (CRP analyzer, blood pressure monitor, digital fever device, ECG device), advanced wearables, and long experience using GPT-4 and o3 for preliminary health assessments. Their findings showed the following: A home finger-stick CRP test measured 130 mg/L (reference <5 mg/L) using an Aidian QuickRead Go analyzer. Body temperature was 39.7°C (Microlife digital fever thermometer), blood pressure was 112/68 mmHg (Microlife), resting heart rate was 58 bpm (Garmin Fenix 6X PRO Solar), and a mobile ECG (KardiaMobile) showed normal sinus rhythm. Based on the clinical presentation, diagnostic data, and context of care unavailability, what is the most likely diagnosis? What should the family consider as the next step in terms of safe, guideline-aligned treatment, assuming human clinical oversight is available remotely?

Based on several minutes of reasoning and analysis of the abovementioned health data, the o3- model extended its former feedback and suggested bacterial tonsillitis as the most likely diagnosis and recommended in-person assessment within 24 h (see Supplementary Material S1). With no access to primary health care and a GP for five days, a licensed physician within the family network examined the patient that evening (physical presence), noting bilateral exudative tonsillar hypertrophy, tender cervical lymphadenopathy, and ongoing fever. The physician received the same health data from the preliminary analysis conducted by the family and the o3 model and concluded that there was a high likelihood of bacterial infection and acute tonsillitis. Phenoxymethylpenicillin (Apocillin) 660 mg four times daily for ten days was prescribed, together with alternating ibuprofen and paracetamol for analgesia. He started with Apocillin Wednesday evening.

**Day 3 (Thursday May 1st).** The patient showed no signs of clinical improvement over the following day. He continued to experience persistent high fever, ongoing sore throat, and significant discomfort when swallowing. Fever a little bit lower at 39.3°C The tonsils remained visibly swollen and inflamed, with pronounced redness and possible exudate. His general condition was unchanged, and the symptoms were affecting his ability to eat, drink, and rest. The lack of response to over-the-counter antipyretics such as paracetamol suggested a more serious underlying infection, potentially requiring further clinical evaluation and targeted treatment.

**Figure 2:**
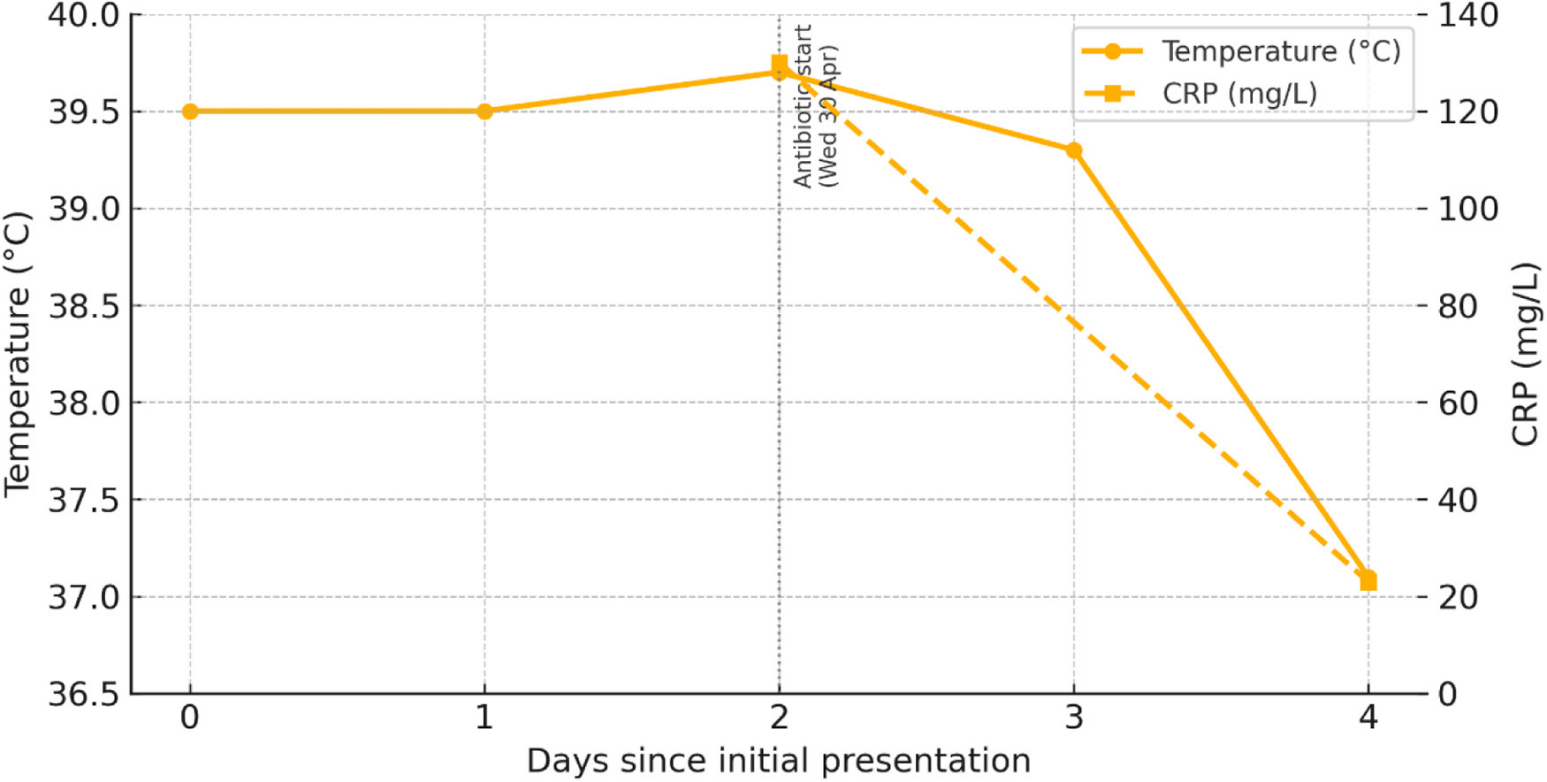
Clinical course: temperature and CRP responses.

**Day 4 (Friday May 2nd).** The Day 4, the patient was afebrile (37.1°C) (see Figure 2), indicating that the fever had resolved. His general condition had improved notably, and he reported only mild discomfort in the throat. Clinical examination showed a reduction in tonsillar swelling and redness. The inflammatory marker C-reactive protein (CRP) had decreased to 23 mg/L (see Figure 2), reflecting a positive response to treatment and a significant reduction in systemic inflammation. The patient was able to eat and drink more comfortably, and no new symptoms had developed.

**Day 5–6 (Saturday 3rd and Sunday 4th May).** The patient recovered gradually and had no signs of illness on Monday 5th May. However, he completed the full 10-day course of penicillin.

No complications were reported at one-week telephone follow-up with the physician in the family network.

## Supplementary evaluation and literature context

### GP capacity and geographic isolation

National registry data and expert reports indicate that access to primary health care and GPs are challenging for citizens located in rural settings—especially when villages are closed down because of landslides, avalanches, etc (2–4). The deficit is most acute in avalanche-prone mountain valleys and Arctic archipelagos where recruiting and retaining doctors is challenging and seasonal avalanches or landslides routinely sever the sole access roads for days at a time (10, 11). Studies such as Harbitz et al. (12) have documented how road closures and high locum turnover contributed to patient safety incidents and delays in acute care. Both research by Solheim et al. (6) and projections from the Norwegian Water Resources and Energy Directorate (7) suggest that such natural events are likely to increase in the coming years.

### Point-of-care CRP testing for pharyngotonsillitis in rural settings

In rural or remote areas without regular access to primary healthcare, CRP testing can be a valuable adjunct to clinical assessment, helping to guide timely management decisions and reduce unnecessary antibiotic use. Evidence from Kara et al. (53) on acute tonsillopharyngitis underscores the potential of combining point-of-care biomarkers with clinical evaluation to improve diagnostic accuracy in such settings. Portable finger-stick analyzers deliver laboratory-grade accuracy up to 200 mg/L and are increasingly deployed by community nurses in remote districts, reducing unnecessary winter travel to outpatient clinics. In the present case, a CRP of 130 mg/L, combined with otherwise normal vitals, supported immediate antibiotic initiation in line with national guidance.

### Large-language-model decision support in hazard-isolated communities

A 2025 systematic review of 73 studies found that AI tools—particularly large-language-model (LLM) chatbots—can improve diagnostic accuracy and patient self-efficacy in primary care when embedded within robust governance frameworks (14). A growing body of evidence points to promising directions (15–20). Rapid reviews echo these possibilities but highlight ongoing concerns about hallucinations and medico-legal accountability (39). Building on this evidence, the Digital Learning Communities Artificial Intelligence Centre (DLCAIC) at the University of Bergen will pilot o3-chatbot across three avalanche-isolated municipalities. A mixed method study will examine if such AI-supported health empowerment affects primary health care quality in rural settings, self-reported health literacy, citizens anxiety during road closures, societal close-downs, etc. In the current case, the o3-chatbot functioned as a *just-in-time* educational aid—reinforcing red-flag criteria and prompting escalation to a licensed physician while leaving diagnostic confirmation and prescribing to the clinician.

### Limitations of this single-case experience

• Generalizability

A single adolescent cannot represent more complex or vulnerable populations.

• Diagnostic certainty

No throat culture or rapid antigen test was available; pathogen typing therefore remains presumptive. The clinical presentation—tender cervical lymphadenopathy, tonsillar exudate, persistent fever ≥39°C, and a C-reactive protein (CRP) of 130 mg/L—strongly suggested a bacterial pharyngotonsillitis. Nevertheless, the absence of a rapid antigen detection test (RADT) or throat culture leaves diagnostic uncertainty regarding the exact pathogen, especially in adolescents where Fusobacterium necrophorum and early peritonsillar abscess (PTA) must be considered alongside group-A Streptococcus (GAS) (40). Cohort studies show that CRP values >100 mg/L have a positive likelihood ratio >5 for bacterial etiology, yet they cannot discriminate reliably between GAS, F. necrophorum, or evolving PTA (41). Empirical phenoxymethylpenicillin was therefore justified on two grounds: (i) CRP ≥100 mg/L combined with Centor-compatible symptoms meets Norwegian and European guidelines (51) for immediate therapy, and (ii) early antimicrobial cover mitigates the risk of suppurative complications when timely throat cultures are unattainable (42). At the same time, high initial CRP warranted safety-netting: follow-up CRP dropped to 23 mg/L within 72 h, confirming treatment response and supporting de-escalation if clinical improvement had lagged. Recent work on CRP velocity (CRPv) underscores this approach; a ≥40 % decline within 48 h predicts favourable outcomes and facilitates antimicrobial stewardship by identifying candidates for early switch to narrow-spectrum oral agents or shortened courses (41). Our protocol therefore balances prompt, guideline-concordant therapy with active reassessment. In future rural implementations, couriered swabs or portable nucleic-acid tests would further refine pathogen-directed therapy and reduce unnecessary antibiotic exposure (52).

• AI reliability, quantitative transparency and risk benchmarking

Unlike many diagnostic-support systems, the o3 LLM returns narrative reasoning rather than calibrated probabilities; it therefore cannot be scored with traditional metrics such as area under the curve or Brier loss in a single-case design. To maintain transparency, we report verbatim (Supplementary Material S1) the model's differential diagnosis, its explicit “red-flag” alerts (e.g., peritonsillar abscess, airway compromise), and the supervising physician's concordant judgement and treatment decision. In lieu of statistical validation, we supply a structured risk-analysis table (See example of this in Table 1) that maps each step of the workflow to plausible failure modes—hallucination, anchoring bias, data-entry error, loss of connectivity—and specifies technical and procedural mitigations (rule-based guardrails, mandatory human review, follow-up CRP, satellite fallback). This approach makes the single case auditable while highlighting where future multi-patient studies should add quantitative performance benchmarks.

• Regulatory, privacy, and liability landscape for LLM-assisted rural triage

**Table 1:** Most likely diagnoses.

Diagnosis	Key features consistent with this case	Tests/findings that can confirm
Streptococcal tonsillitis (GAS)	Sudden high fever, severe odynophagia; erythematous tonsils often with exudate; CRP frequently 50–150 mg/L.	Rapid antigen detection test or throat culture/PCR for Streptococcus pyogenes.
Peritonsillar abscess	Often evolves from tonsillitis; unilateral worsening pain, “hot potato” voice, trismus; may have uvular deviation; CRP often >100 mg/L.	Clinical examination ± ultrasound/CT; ENT assessment; purulent aspiration confirms.
Epstein–Barr virus (mononucleosis)	Pharyngitis, high fever, marked fatigue; pronounced tonsillar hypertrophy and cervical lymphadenopathy; CRP usually <100 mg/L but can be elevated.	Heterophile antibody (Monospot) and/or EBV serology; atypical lymphocytes on blood smear.
Influenza or SARS-CoV-2 with secondary bacterial tonsillitis	Initial viral prodrome; on days 3–5 a new fever spike with worsening throat pain; CRP then typically >100 mg/L.	PCR for influenza/SARS-CoV-2 plus throat culture.

Note: This table is informational and not a substitute for clinical judgment. CRP, C-reactive protein; GAS, group A streptococcus; ENT, ear, nose, and throat; EBV, Epstein–Barr virus; PCR, polymerase chain reaction.

The workflow we describe processes special-category health data, so any real-world deployment must rely on the clinical-care exception in GDPR Article 9(2)(h), which permits processing when it is “necessary for medical diagnosis or the provision of health care” under professional secrecy obligations (43). Standing alone, however, GDPR does not address algorithmic safety; that gap is now filled by the EU Artificial Intelligence Act—Regulation (EU) 2024/1689 (50). Published in the *Official Journal* on 12 July 2024 and in force since 1 August 2024, the Act imposes tiered duties on providers and deployers of AI systems. Governance rules and transparency duties for general-purpose AI (GPAI) models begin 2 August 2025, while full obligations for high-risk AI systems (including diagnostic decision support in health care) follow on 2 August 2026 (44, 45). A July 2025 Commission statement confirmed that these milestones will not be delayed despite industry pressure. When an LLM is integrated into clinical workflow, it may also qualify as medical-device software under Regulation (EU) 2017/745 (MDR). Under Rule 11, diagnostic or therapeutic decision-support software is at least Class IIa unless human oversight is demonstrably “systematic and prompt”; higher classes apply as patient risk increases (46). MDCG 2021-24 guidance gives practical examples and stresses documentation of the “human-in-the-loop” safeguard for triage chatbots (47). Our design therefore keeps the physician as final decision-maker, researchers logs every AI recommendation, and allows manual override—controls that both the AI Act and MDR recognise as risk-mitigating. Together, GDPR Article 9, the AI Act's phased obligations, and MDR Rule 11 define a clear but demanding compliance path: privacy impact assessment, GPAI provider documentation by mid-2025, and notified-body conformity assessment for any Class IIa/IIb software used beyond mere information provision.

• Location

The patient was located in a city (and not in a rural setting). Still, there were 5 days waiting time for access to a GP for this patient.

• Digital health devices and health hubs

People in rural contexts do not necessarily have access to emergency clinics or private general practitioners, nor do they have the kind of digital health equipment described in this case—or a physician within their family network. Therefore, a viable solution is to establish health hubs in sparsely populated areas where such low-cost diagnostic tools and AI support are available to those who need them. Combined with digital access to a human in the loop—that is, a physician via video link—this approach can help realize the same kind of health empowerment illustrated by this case.

### Implications for future primary health care models

**Figure 3:**
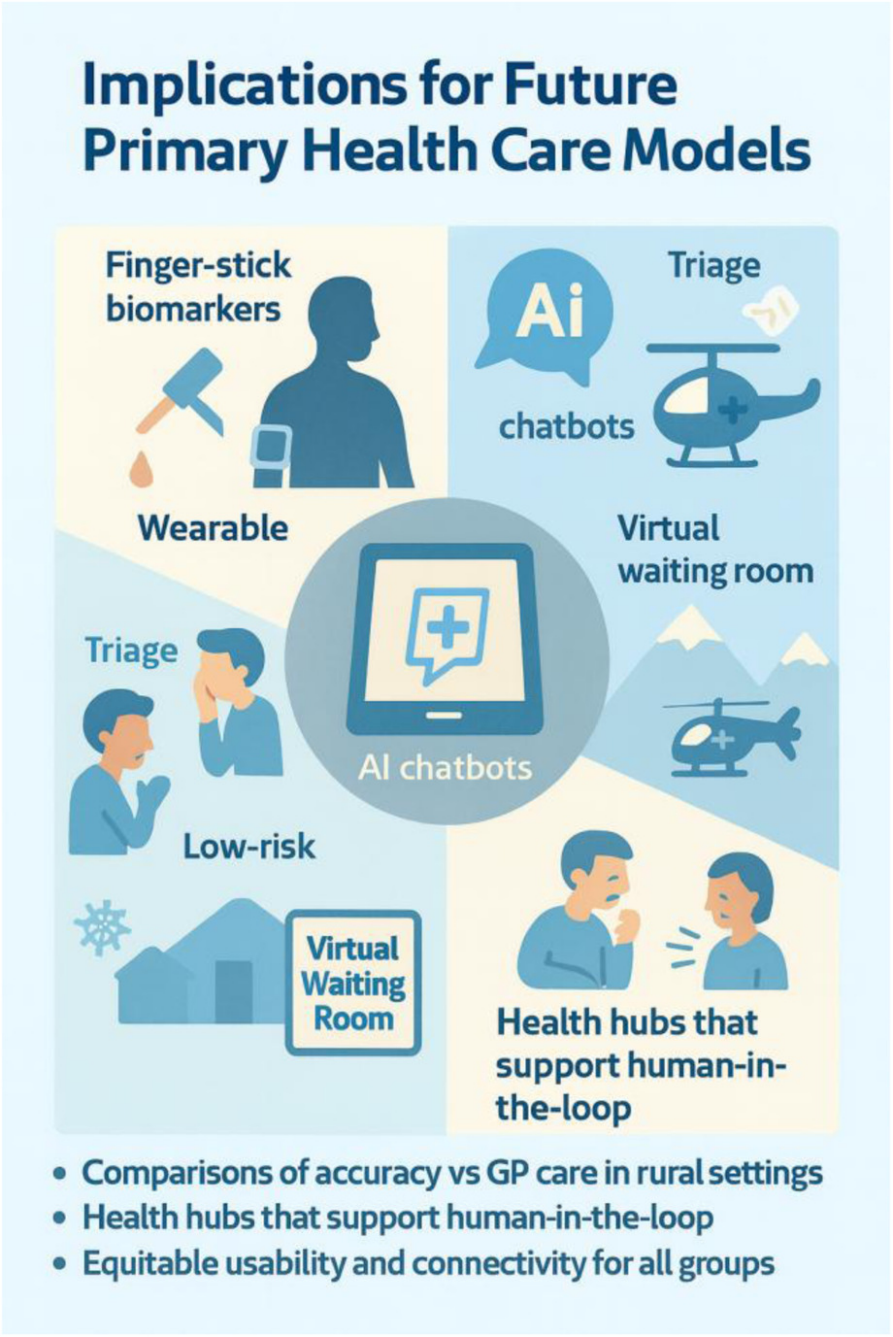
AI-supported health empowerment in rural communities.

Integrating finger-stick biomarkers, wearable-sensor feeds and guideline-trained o3 chatbots (based on RAG and CoT) can create a “virtual waiting room” for rural communities and landslide and avalanche isolated communities: low-risk sore throats and self-limiting infections are counselled and monitored at home, and winter-road helicopter lifts are reserved for higher-acuity patients. Implementation science should now focus on: (a) Direct comparisons of accuracy and safety between AI-supported self-care pathways and standard general practitioner or out-of-hours services in rural Norway; (b) Health hubs with video link and telemedicine with GP to secure human-in-the-loop in rural settings (c) equitable usability among elderly, minority groups and low-income residents who often face patchy connectivity and digital-literacy gaps; and (d) co-design of governance frameworks that satisfy GDPR (43), the forthcoming EU AI Act (42) and Norway's 2030 Digital-Health Strategy (48). Figure 3 summarises how our proposed model could reshape rural primary care. In the upper-left quadrant, home finger-stick biomarkers and wearable sensor streams feed an AI chatbot trained with retrieval-augmented, chain-of-thought prompting. The chatbot performs first-line triage, directing low-risk sore-throat cases to a “virtual waiting room” for remote monitoring for low risk patient (lower-left), while reserving scarce helicopter or winter-road transfers for high-acuity patients (upper-right). The lower-right quadrant highlights the core message of the study illustrated by community “health hubs” that maintain a human-in-the-loop: a video-linked GP validates AI advice and prescribes when needed. Finally, the lower-left bullet list distils the next research and implementation priorities—head-to-head accuracy studies against standard GP care, hub deployment logistics, equitable usability for digitally-marginalised groups, and co-designed governance compliant with GDPR and the forthcoming EU AI Act. Together, the graphic translates the narrative of our study into an at-a-glance roadmap for future service design in avalanche-isolated regions. The figure underscores the study's core claim: when connectivity, clinician oversight, and governance safeguards are in place, AI support can shift elements of diagnosis and follow-up closer to the patient, thereby boosting self-efficacy, health empowerment and resilience in avalanche-isolated rural communities.

### Methodological implications

Through a series of case studies, we have pretested the capabilities and reliability of GPT-4 and o3 in educational and healthcare contexts. The o3 model was used in this case because of its exceptionally high performance on the ARC-AGI benchmark—a comprehensive test designed to evaluate advanced reasoning and general intelligence in AI systems (49). Scoring highly on this benchmark indicates that the model is capable of handling complex, real-world tasks that require nuanced understanding and multi-step reasoning. In the context of this case study, the o3 model's strong ARC-AGI results support its use as a decision-support tool for evaluating clinical symptoms and suggesting plausible next steps, especially when human oversight is integrated. Based on this knowledge base, we take a step further by testing these technologies in an authentic patient case set in a fictitious location. Given the sensitive nature of health data and the regulatory constraints imposed by GDPR and broader privacy concerns, we recommend that similar initiatives adopt DLCAIC's pretesting strategy. Conducting carefully designed, small-scale and authentic single case studies in ethically sound, simulated contexts allows for early insight into both technological functionality and contextual risks. This methodological approach offers a responsible and adaptable framework for evaluating AI applications before scaling up larger, more complex clinical studies.

## Conclusion

The research question of this study was: *To what extent, and in what ways, might low-cost diagnostics and AI-supported decision-making contribute to health empowerment in managing severe tonsillitis in rural areas with limited access to primary care?* This single case experience shows that AI supported health empowerment with *symbiotic intelligence* (24)—the coordinated use of home biomarkers, wearable data streams, and an o3 chatbot under human clinical oversight—can deliver timely, guideline-concordant care for acute bacterial tonsillitis when rural location, landslide and avalanche-related road closures and GP shortages make conventional treatment impossible. Moreover, this form of digitally supported health empowerment might have the potential to strengthen primary healthcare in new ways for residents of sparsely populated areas, by bridging care gaps and enhancing local health resilience through low-cost tools, AI support, and remote clinical supervision. But as several studies show (21, 22), we are still in an early stage of such AI-supported health care, where the knowledge base is limited and many ethical challenges need to be addressed. These must be prioritized in the years to come before the full-scale implementation of AI in rural areas.

## Data Availability

The raw data supporting the conclusions of this article will be made available by the authors, without undue reservation.
