# Supplementary material for: Artificial intelligence and health empowerment in rural communities and landslide- or avalanche-isolated contexts: real case at a fictitious location
Source: Front Digit Health. 2025 Aug 26;7:1655154. doi: 10.3389/fdgth.2025.1655154 (PMC12417106; doi:10.3389/fdgth.2025.1655154)
Supplement: Supplementary file 1 [file Datasheet1.pdf]

Supplementary file

A. The first preliminary and general prompt

A 16-year-old boy is sick with body aches, a high fever, and severe throat pain. His resting heart rate and blood pressure are normal, but his CRP is 130 mg/L. Could you try to make a diagnosis based on this information?

A condensed response from o3, part 1: *The numbers primarily point to acute bacterial tonsillitis (most likely streptococcal), but an early peritonsillar abscess must also be ruled if the CRP is high and the throat pain is severe. A medical evaluation with CRP, a throat swab (and possibly imaging) is necessary for a definitive diagnosis and appropriate treatment (OpenAI, 2025b).*

B. The second prompt is based on a more precise description:
You are a medically informed assistant helping to evaluate a 16-year-old boy who returned home from school with a high fever (39.5 °C). He presents with a sore throat, swollen red tonsils, difficulty swallowing, and bad breath. Paracetamol (2 × 500 mg every 5 hours) has provided little relief. He lives in a remote area where access to a doctor is currently unavailable due to road closures. Based on this clinical picture and context, what is the most likely diagnosis, and what would be an appropriate next step in management?

A condensed response and preliminary analysis of the symptoms by using o3*: The data mainly point to acute bacterial tonsillitis (most likely streptococcal), but an early peritonsillar abscess must also be ruled out, especially if the CRP is high and severe throat pain persists. A medical assessment with CRP and throat sampling (and possibly imaging) is necessary for a definite diagnosis and appropriate treatment (OpenAi, 2025b).*

C. The third prompt is based on *chain-of thought prompting*

As mentioned, you are a medically informed assistant helping to evaluate a 16-year-old boy who returned home from school with a high fever (39.7 °C), sore throat, swollen and red tonsils, difficulty swallowing, and bad breath. Paracetamol (2 × 500 mg every 5 hours) has provided little relief. The family lives in a remote, avalanche-prone mountain area where road closures have cut off access to healthcare. Repeated attempts to contact the local GP clinic were unsuccessful due to fully booked schedules and physician absences, and the earliest available appointment was five days away. The family has access to point-of-care diagnostic tools at home (CRP analyzer, blood pressure monitor, digital fever device, ECG device), advanced wearables, and long experience using GPT-4 and o3 for preliminary health assessments. Their findings showed the following: A home finger-stick CRP test measured 130 mg/L (reference < 5 mg/L) using an Aidian QuickRead Go analyzer. Body temperature was 39.5 °C (Microlife digital fever thermometer), blood pressure was 112/68 mmHg (Microlife), resting heart rate was 58 bpm (Garmin Fenix 6X PRO Solar), and a mobile ECG (KardiaMobile) showed normal sinus rhythm. Based on the clinical presentation, diagnostic data, and context of care unavailability, what is the most likely diagnosis? What should the family consider as the next step in terms of safe, guideline-aligned treatment, assuming human clinical oversight is available remotely?

A condensed response from o3, part 3:

"Note! I am not a physician, and the information below can never replace a clinical assessment by qualified healthcare personnel. If the boy's overall condition worsens, he develops shortness of breath, has great difficulty swallowing, becomes very lethargic, or you are in doubt — contact the out-of-hours medical service (tel. 116 117 in Norway) or dial 113 immediately.

Brief summary

- **Symptoms:** Generalised body aches, high fever, pronounced sore throat.
- **Objective findings:** Resting pulse and blood pressure are normal, but **CRP 130 mg/L** (high).
- **Interpretation:** A CRP > 100 mg/L strongly suggests a *bacterial* cause, but differential diagnoses must be considered.

Most Likely Diagnoses

Diagnosis	Key Features Consistent with This Case	Tests/Findings That Can Confirm
Streptococcal tonsillitis (GAS)	Sudden high fever, severe odynophagia; erythematous tonsils often with exudate; CRP frequently 50–150 mg/L.	Rapid antigen detection test or throat culture/PCR for Streptococcus pyogenes.
Peritonsillar abscess	Often evolves from tonsillitis; unilateral worsening pain, “hot potato” voice, trismus; may have uvular deviation; CRP often >100 mg/L.	Clinical examination ± ultrasound/CT; ENT assessment; purulent aspiration confirms.
Epstein–Barr virus (mononucleosis)	Pharyngitis, high fever, marked fatigue; pronounced tonsillar hypertrophy and cervical lymphadenopathy; CRP usually <100 mg/L but can be elevated.	Heterophile antibody (Monospot) and/or EBV serology; atypical lymphocytes on blood smear.
Influenza or SARS-CoV-2 with secondary bacterial tonsillitis	Initial viral prodrome; on days 3–5 a new fever spike with worsening throat pain; CRP then typically >100 mg/L.	PCR for influenza/SARS-CoV-2 plus throat culture.

- *Note.* This table is informational and not a substitute for clinical judgment. Abbreviations: CRP = C-reactive protein; GAS = group A streptococcus; ENT = ear, nose, and throat; EBV = Epstein–Barr virus; PCR = polymerase chain reaction.

Viral vs. bacterial pointers

- **CRP < 50 mg/L** → usually viral.
- **CRP 50 – 100 mg/L** → indeterminate.
- **CRP > 100 mg/L** → strongly points to bacterial infection (e.g. streptococci, abscess, bacterial super-infection).

Recommended follow-up

1. **Clinical examination by a doctor the same day**
2. **Laboratory tests**
3. **Treatment (depending on findings)**
4. **Symptom relief at home**
5. **When to seek emergency help**

Conclusion

The data mainly point to **acute bacterial tonsillitis (most likely streptococcal)**, but an early **peritonsillar abscess** must also be excluded because of the high CRP and severe throat pain. A medical assessment with throat sampling (and possibly imaging) is necessary for a definite diagnosis and appropriate treatment (OpenAi, 2025b).
